# Trastuzumab and target-therapy side effects: Is still valid to differentiate anthracycline Type I from Type II cardiomyopathies?

**DOI:** 10.1080/21645515.2015.1125056

**Published:** 2016-02-02

**Authors:** Gennaro Riccio, Carmela Coppola, Giovanna Piscopo, Immacolata Capasso, Carlo Maurea, Emanuela Esposito, Claudia De Lorenzo, Nicola Maurea

**Affiliations:** a Istituto Nazionale per lo Studio e la Cura dei Tumori “Fondazione Giovanni Pascale” –IRCCS – Naples, Italy; b CEINGE Biotecnologie Avanzate, Naples, Italy; c Dipartimento di Medicina Molecolare e Biotecnologie Mediche, Università Federico II, Naples, Italy

**Keywords:** anthracyclines, cardiotoxicity, cancer therapy, immunotherapy, trastuzumab

## Abstract

The improvement in cancer therapy and the increasing number of long term survivors unearth the issue of cardiovascular side effects of anticancer treatments. As a paradox in cancer survivors, delayed cardiotoxicity has emerged as a significant problem. Two categories of cardiotoxic side effects of antineoplastic drugs have been previously proposed: Type I cardiotoxicity, defined as permanent cardiotoxicity, is usually caused by anthracyclines; Type II cardiotoxicity, considered as reversible cardiotoxicity, has been mainly related to monoclonal antibodies. The cardiotoxicity of antibodies has been associated to trastuzumab, a humanized anti-ErbB2 monoclonal antibody currently in clinical use for the therapy of breast carcinomas, which induces cardiac dysfunction when used in monotherapy, or in combination with anthracyclines. Furthermore, recent retrospective studies have shown an increased incidence of heart failure and/or cardiomyopathy in patients treated with trastuzumab, that can persist many years after the conclusion of the therapy, thus suggesting that the side toxic effects are not always reversible as it was initially proposed. On the other hand, early detection and prompt therapy of anthracycline associated cardiotoxicity can lead to substantial recovery of cardiac function. On the basis of these observations, we propose to find a new different classification for cardiotoxic side effects of drugs used in cancer therapy.

## ABBREVIATIONS

BC: breast carcinomas
CHF: congestive heart failure
LVEF: left ventricular ejection fraction
HF: heart failure
CM: cardiomyopathy
LVESV: left ventricular end systolic volume
LVEDD: left ventricular end diastolic dimension; RS, radial strain

## Introduction

It has been estimated that in January 2014 in USA about 14,5 million people with a history of cancer were still alive,^1^ and that this number will increase to nearly 18 million within 2022.^2^ Moreover about 50% of cancer survivors will die by direct effects of cancer whereas nearly 33% of them for cardiotoxic side-effects of cancer therapy (National Health and nutrition Examination Surveys (NHANES) American Association for Cancer Research (AACR) Annual Meeting, March 31-April 4 2012).

The improvement in cancer therapy and the increasing number of long-term survivors, unearth the relevant issue of cardiovascular side effects of anticancer treatment. ^3^ As a paradox in cancer survivors, delayed cardiotoxicity has emerged as a significant problem. Two categories of cardiotoxic side effects of antineoplastic drugs have been proposed by Ewer.^4^ Type I and Type II cardiotoxicity.

Type I cardiotoxicity, defined as permanent cardiotoxicity, is usually caused by anthracyclines. Type II cardiotoxicity, considered as reversible cardiotoxicity, has been mainly related to monoclonal antibodies or tyrosine kinase inhibitors.^4^

The mechanism underlying anthracycline cardiotoxicity is well described in literature and includes cell damage due to radical free formation and oxidative stress.^5^

The cardiotoxicity of anti-ErbB2 antibodies has been associated to trastuzumab, a humanized anti-ErbB2 monoclonal antibody, currently in clinical use for the therapy of BC.^6^ Trastuzumab was initially shown to prolong the survival of women with HER-2-positive advanced BC.^7^ In 2005, landmark adjuvant studies demonstrated that adjuvant Trastuzumab either after, or in combination with, chemotherapy reduced the risk of relapse by approximately 50% and the risk of death by 33% for women with HER-2 positive early BC.^8^

However, cardiac toxicity was recognized as an important side effect at an early stage in the development of Trastuzumab, manifested as symptomatic CHF or asymptomatic LVEF decline,^9^ that in some cases can cause severe cardiac insufficiency and even death.^10^

Large-scale clinical studies with Trastuzumab, have shown that up to 7% or 28% of patients suffer from cardiac dysfunction when Trastuzumab is used in monotherapy, or in combination with anthracyclines, respectively.^7,11^ A similar increase of cardiotoxic side effects was observed when Trastuzumab was combined with paclitaxel, as post hoc analyses revealed up to 11% cardiotoxicity in patients receiving trastuzumab on top of paclitaxel compared with only 1% to 4% in those who received paclitaxel alone.^12^

Recently independent studies have shown an increased incidence of HF and/or CM in patients treated with trastuzumab in monotherapy (up to 30%) or in combination with anthracyclines (up to 40%), particularly in elderly breast cancer patients with a history of other diseases.^13-15^

In these studies an interesting aspect has been evidenced: the trastuzumab related cardiotoxic side effects progressively increased during 3–5 years after the end of treatment with trastuzumab, thus suggesting that the cardiotoxicity onset could occur even post treatment and the patients were not appropriately subjected to follow up inspections.

In agreement with these observations, ICARO Network showed an increased trastuzumab-related cardiotoxicity (up to 38%) in elder patients (age > 60 years) according with data previously described.^13-15^ Furthermore, the ICARO Network studies have pointed out a higher percentage of trastuzumab-related cardiotoxicity associated to cardiovascular risk factors as well as in patients affected by cardiac diseases such as heart failure or hypertension.^16,17^

Cardiotoxicity induced by Trastuzumab had long been considered reversible as it has been supposed that the withdrawal of the antibody allows for the return of function of the ErbB2 cardiomyocyte survival pathway and reversal of EF decline, in contrast to the permanent myocyte dysfunction induced by anthracyclines.

The mechanism of cardiotoxic effects of trastuzumab is not completely clarified, but it has been attributed to blockade of HER-2 signaling in cardiac myocytes as trastuzumab interferes with ErbB2/ErbB4 heterodimerization induced by Neuregulin1^18^ and its signaling pathways essential for myocyte function and repair for heart injury.^19^

However recent retrospective studies suggest that the cardiotoxic side effects of Trastuzumab should be carefully reconsidered as they can persist many years after the conclusion of the therapy, thus strongly suggesting that they are not always reversible as it was initially proposed.^4^ Furthermore, several *in vitro* and *in vivo* studies in human cardiac cell cultures and in mice, respectively, clearly indicate that Trastuzumab induces apoptosis of cardiomyocytes thus leading to irreversible cell death.^20,21^

## Incidence of heart failure and/or cardiomyopathy after trastuzumab therapy

Several studies report on risk prediction model based on echocardiography imaging analyses in patients treated with anthracyclines or trastuzumab,^22,9^ but also epidemiological analyses of the women treated with anthracyclines and/or trastuzumab in adjuvant therapy ^13,14^ have been used to extrapolate a risk prediction model for HF and CM based on the history of patients.^23^

Chen J et al. observed, in a cohort of 45537 women with early stage breast cancer, that the cumulative incidence of HF or CM was higher for patients treated over three years with trastuzumab alone (32.1%) and trastuzumab with anthracyclines (A+T) (41.9%) compared with patients who received no adjuvant therapy (18.1%). In secondary analyses carried out by evaluating HF and CM separately, the combined treatment of anthracyclines and trastuzumab was associated with HF alone and CM alone compared with no adjuvant therapy in their models. Trastuzumab in the absence of anthracycline therapy was borderline significantly associated with HF alone, but was clearly associated with CM alone compared with no adjuvant therapy. Furthermore, compared with patients who received no adjuvant chemotherapy or trastuzumab, the use of trastuzumab was associated with an absolute 14% higher incidence rate for HF or CM.^14^

Another study reports on a retrospective analysis of a cohort of 12500 women diagnosed with breast cancer and treated by adjuvant therapy.^13^ In this study the authors evaluated the incidence of HF/CM risk associated with chemotherapy and/or trastuzumab use. The cumulative incidence data are well summarized by Bowles et al.^13^ as reported in table I, which shows that cumulative incidence of HF and CM in patients treated with anthracyclines enhanced with increasing age. The incidence of HF and CM in patients treated with anthracyclines plus trastuzumab also increased with age advancement. Although a few number of patients received trastuzumab-based therapy without anthracyclines it is possible to point out a higher cumulative incidence of HF/CM in older women (31.5% per age > 75 years; Table 1),^13^ consistent with the observation of other authors.^15,23^

**Table 1.** Cumulative incidence of heart failure and/or cardiomyopathy and hazard ratio in women with invasive breast cancer over 5 years by adjuvant chemotheraphy group.^1,3^

Cumulative incidence of HF/CM and hazard ratio over 5 years of treatment		
All ages		
Treatment	Cumulative incidence (%)	Hazard ratio (1.00 no chemotherapy)
Anthracycline only	4.3	1.40
Trastuzumab only	12.1	4.12
Anthracycline + Trastuzumab	20.1	7.19
Age < 55years		
Anthracycline only	1.2	2.52
Trastuzumab only	7.1	15.45
Anthracycline + Trastuzumab	7.5	16.36
Age 55–64 years		
Anthracycline only	2.9	1.61
Trastuzumab only	17.7	10.76
Anthracycline + Trastuzumab	11.4	6.69
Age 65–74 years		
Anthracycline only	6.2	1.22
Trastuzumab only		
Anthracycline + Trastuzumab	35.6	8.34
Age ≥75 years		
Anthracycline only	10.6	0.76
Trastuzumab only	31.5	2.57
Anthracycline + Trastuzumab	40.7	3.54

On the contrary, the hazard ratio for HF and CM (the incidence of HF and CM in treated patients with respect to untreated control population) associated with anthracyclines seems to be higher in younger patients as it is statistically significant among women younger than 55 years but not among older women. Similarly the hazard ratio associated with trastuzumab treatment is higher in younger women (< 64 years) with respect to older women (> 74 years). Bowles et al. report an hazard ratio of 15.46 or 10.16 in women younger than 55 years and women aged 55–64 years respectively, 5 years since start of treatment. In elder women a hazard ratio of 2.57 was reported. These hazard ratios suggest a fourfold increase in the risk of HF or CM among women treated with trastuzumab.

Trastuzumab toxicity is not only directly related to the presence of other risk factors, as in a more accurate analysis Bowles et al. reported the hazard ratio corrected by excluding women with comorbidities, and the results did not change significantly. The overall risk of incidence of HF or CM was increased in women treated with anthracyclines, but it was even greater in women treated with trastuzumab.

Moreover some authors discuss how trastuzumab, classified as type II cardiotoxic agent, can also trigger irreversible cardiotoxicity,^24^ and other studies have shown that the incidence of trastuzumab-related cardiotoxicity observed in the clinical setting is higher than what reported in clinical trials.^25^

Altogether these are important observations because they strongly indicate that side effects of trastuzumab not only affect women during treatment, but they also occur many years after the treatment as a delayed cardiotoxicity. The mechanisms of cardiotoxicity induced by anthracycline or by trastuzumab may explain the difference in the risk of HF or CM observed after cancer therapy with these antineoplastic drugs.

## Mechanisms of trastuzumab-induced cardiotoxicity

**Figure 1.**
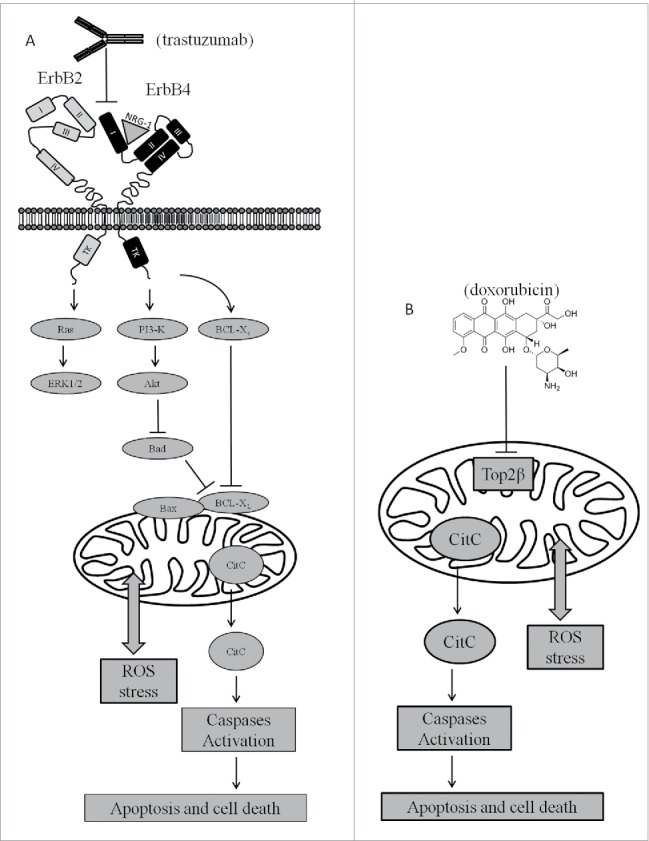
(A) trastuzumab inhibits ErbB2-ErbB4 pathway triggering adverse effects similarly to doxorubicin treatment. (B) Oxidative stress and citochrome C release cause adverse effects in cardiomyocytes following Doxorubicin treatment.

Several studies indicate that ErbB2 has an important role in heart development and function. In cardiac tissues ErbB2 works as a co-receptor for another ErbB receptor tyrosine kinase family member, ErbB4 and its peptide ligand neuregulin 1 (NRG1).^26^ NRG1 binds to ErbB4 on cardiomyocytes and promotes ErbB4/ErbB2 heterodimerization, which triggers autophosphorylation of the heterodimers, increases tyrosine kinase activity and induces ERK-MAPK and PI3K-Akt pathways,^27,28^ thus promoting cardiomyocyte proliferation, contractile function and survival.^19^ Furthermore, block of HER2 has been correlated with a change in the antiapoptotic/proapoptotic proteins ratio, due to a downregulation of BCL-XL (see Fig. 1), an antiapoptotic protein, and an upregulation of BCL-XS, a proapoptotic protein.^29^ As they are key mediators in mitochondrial function and apoptosis, a shift in their ratio toward proapoptotic proteins is correlated with mitochondrial dysfunction, which finally leads to cardiomyocyte death.^30,31^

Even though trastuzumab prolongs the survival of women with Her2 positive breast cancer, its cardiotoxic side effects are important. The initial hypothesis has been made that trastuzumab-associated cardiotoxicity is related to the inhibition of the neuregulin-1 activated pathway.^32^ This pathway seems to be stimulated when myocardium addresses adverse stresses such as anthracycline therapies.^33^ However the last epidemiological studies indicated that trastuzumab toxicity is not only related to the presence of other risk factors as an increase in the risk of HF or CM among women treated with trastuzumab only has been observed.^13,14^

Another possible mechanism for trastuzumab-associated cardiotoxicity is represented by mithocondrial dysfunction and distruption of cellular energetic due to the altered BCL-X_L_/BCL-X_S_ ratio, which likely leads to loss of mitochondrial membrane integrity with multiple major deleterious intracellular consequences including loss of electron transport, generation of free radicals, uncoupling of oxidative phosphorylation which leads to ATP reduction, release of pro-apoptotic proteins such as cytochrome c.^34,35^

Trastuzumab-associated cardiotoxicity was confirmed by findings obtained *in vitro* on cultures of cardiac cells and *in vivo* on animal models. *In vivo* studies in mice have shown that trastuzumab treatment significantly affects both functional and structural properties of the heart. It altered the expression of genes involved in cardiac functions, adaptability to pressure, vasodilatation and contractility (Myl4, Myl7, Mhy1, Rxfp1, Ttn, Nppa, Acta1),^20,21^ those involved in regulation of calcium and sodium processing (FGF12 and Sln), and those genes that regulate DNA repair, mitochondrial function and apoptosis (Fbxl7 and Atf3).^20^ Moreover Trastuzumab raised myocardial levels of 4-hydroxynonenal (4-HNE) and 3-nitrotyrosine (NT), products of oxidative and nitrative stess,^20^ which can trigger mitochondrial dysfunction and other damages in cardiac tissue.^36^

Cardiac damage has been observed also by electron microscopy imaging. Trastuzumab treatment causes cardiomyocyte ultrastructure alteration, associated with ultrastuctural damages of heart tissues in mice. In particular, alterations in intermitochondrial distance, thickness of myofibers and number of mitochondria have been observed.^20^ It is noteworthy that both oxidative stress and structural alterations have been similarly found to be the main pathways in doxorubicin related cardiotoxicity, as described above.

**Figure 2.**
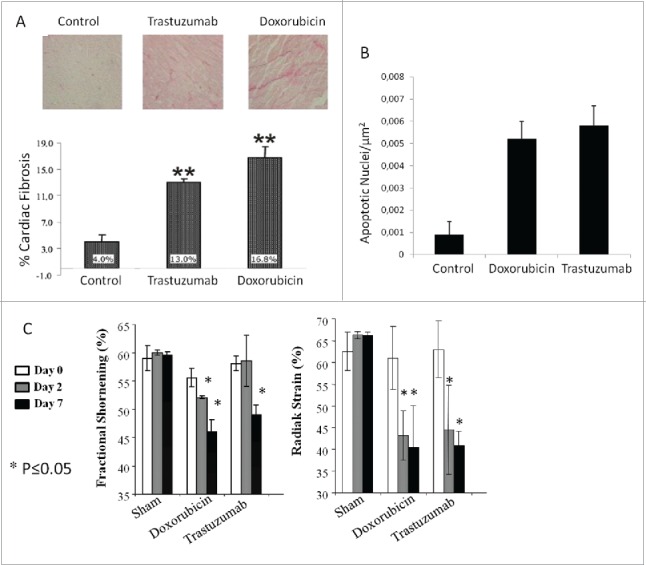
(A) Analysis of cardiac fibrosis (A1: representative photomicrographs of LV sections from mice treated with trastuzumab or doxorubicin; A2: quantification of interstitial fibrosis expressed as relative percentage). (B) TUNEL assay of cardiac tissue sections from mice treated with trastuzumab or doxorubicin. (C) effects on cardiac functions of doxorubicin or trastuzumab.^21,44^.

Moreover, in trastuzumab-treated mice it has been shown an activation of apoptotic pathways, such as PARP cleavage, caspase 3/7 activation and increase of Bax/BCL-X_L_ ratio.^20,37^ The activation of apoptotic pathways in cardiomyocytes of mice treated with trastuzumab has been supported also by other experimental evidences. The number of the apoptotic cells and the extent cardiac fibrosis in cardiac tissue was found to be significantly increased in mice treated with trastuzumab with respect to control untreated mice (see Fig. 2).^21,38^ In these studies trastuzumab was found to affect cardiomyocytes in treated mice likewise doxorubicin.

Functional alterations of heart in treated mice were also detected as trastuzumab was found to significantly reduce fractional shortening by increasing left ventricular end-diastolic dimension (LVEDD).^21^ Moreover Trastuzumab, in a fashion similar to doxorubicin, reduces radial strain (RS) before the alteration of fractional shortening (FS) after only two days of treatment.^18^

**Figure 3.**
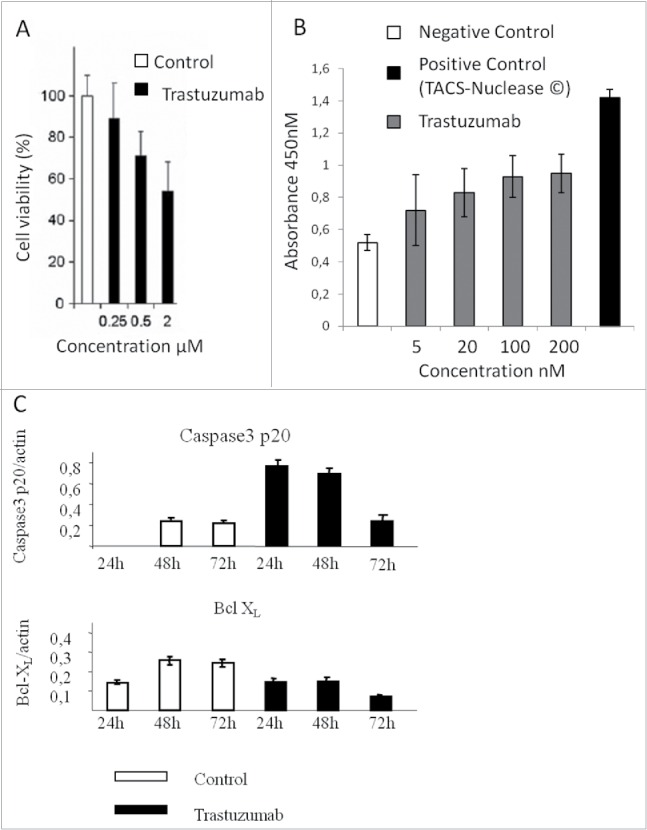
(A) Effects of trastuzumab on cardiac cells. Dose response tests of human fetal cardiomyocytes treated for 24 h with trastuzumab (black). (B) DNA fragmentation as induced by trastuzumab treatment in H9C2 cells. (C) Caspase 3 activation and BCL-X_L_ downregulation in H9C2 cells treated with trastuzumab at different times. ^21^

In *in vitro* assays Trastuzumab was found to significantly reduce cell viability of cardiomyocytes.^21,39^ Cell viability reduction was due to induction of apoptosis, as shown in Figure 3. Trastuzumab was found capable to significantly increase the levels of activated caspase3 and to reduce the levels of Bcl-X_L_ in cardiomyocytes, thus inducing apoptosis.^21^

In these *in vitro* studies it has been also confirmed that the cardiotoxic effects of trastuzumab are also based on its ability to prevent the assembly of NRG-1/ErbB2/ErbB4 complex, required for cardiomyocyte survival. Indeed trastuzumab treatment was capable of inhibiting ligand-induced cell proliferation, thus affecting not only the basal cardiomyocyte survival but also the ligand-induced ErbB2/ErbB4 heterodimerization signaling pathway downstream ^39^ needed for cell growth and functionality. These findings may provide the molecular basis for trastuzumab-related cardiotoxicity.

## Mechanisms of anthracycline-associated cardiotoxicity

Doxorubicin is a highly effective anthracycline but its clinical use is compromised by the development of a severe form of cardiomyopathy and heart failure. The mechanisms of the doxorubicin-related cardiotoxicity have been extensively studied and two main pathways have been proposed. First, doxorubicin has been supposed to induce cardiotoxicity by ROS formation,^40^ as confirmed by several studies.^5,41,42^ Second, doxorubicin, in cancer cells, binds both to DNA and to topoisomerase-II (Top2) to form a ternary complex which triggers cell death.^43^ It has been shown that the doxorubicin-top2β complex in cardiomyocytes plays a key role to induce changes in mitochondrial membrane potential, mitochondrial function and morphology.^44^ Mitochondrial dysfunction causes an increase in the Bax:Bcl-2 ratio, cytochrome C release leading to caspase-3 activation and following apoptosis ^45^ (see Fig. 1). Moreover the doxorubicin-top2β complex is needed to induce cardiac dysfunction in mice, as in cardiomyocyte-specific Top2β-deleted mice doxorubicin does not induce alterations in LVESV, diastolic volume (LVEDV) and ejection fraction.^44^ All these effects induced by doxorubicin may explain the permanent cardiac damage and the following arise of cardiomyopathy or heart failure in treated patients.

## Concluding remarks

In the past few decades, novel anticancer drugs have been developed that effectively induce tumor regression, thus prolonging patient's survival. However, cardiovascular side effects of these new drugs can lead to therapy related heart failure. Cardiac side effects of anti-Her2 anticancer agents, such as trastuzumab or tyrosine kinase inhibitors, were totally unexpected.^39^ Initially trastuzumab has been shown to induce severe cardiotoxic effects particularly when used in combination with anthracyclines (up to 28% of treated patients).^7^ Following these observations, treatment regimens were altered, initiating treatment with trastuzumab only after completion of anthracycline therapy. Although these measures reduced the incidence of symptomatic chronic heart failure to less than 5%, asymptomatic cardiac dysfunction still occurs in 14% of patients receiving the trastuzumab plus anthracycline and in 7% receiving trastuzumab alone.^12^

Thus, the initial hypothesis has been made that trastuzumab-associated cardiotoxicity is related to the inhibition of the NRG1 activated pathway,^32^ which directly promotes cardiomyocyte survival via ErbB2/ErbB4 heterodimerization, in response to oxidative stress due to anthracycline therapies. However this hypothesis does not explain exhaustively the trastuzumab related cardiotoxicity, observed in patients treated in monotherapy, which is even increased in elder patients becoming life threatening for patients over 70 years old.^15,46^

Our studies have demonstrated that trastuzumab directly induces damage in cardiomyocytes both *in vitro* and *in vivo*.^20,21,37,39^ Moreover human cardiomyocytes undergo several stresses during lifespan: hemodynamic, hypoxic or oxidative stress and in these cases ErbB2/ErbB4 heterodimerization pathway triggered by Neuregulin is activated.^28^

 Systolic and diastolic dysfunctions, asymptomatic myocardial ischemia or undiagnosed myocarditis are more frequently in the elderly. Thus it is likely that ErbB2/ErbB4 pathway is chronically activated in these conditions and its blockage by trastuzumab treatment may empathize trastuzumab-related cardiotoxicity in elder patients with undiagnosed heart diseases.

This may explain the increased trastuzumab-associated cardiotoxicity in elderly or in patients with other risk factors related to old age such as the higher incidence of systo-diastolic left ventricular dysfunction or chronic myocardial ischemia in diabetic patients.^15^

Some data suggest that cardiotoxicity in patients treated with anthracyclines is not necessarily irreversible but it may become reversible if early diagnosed.^47^ Furthermore the reversibility of left ventricular dysfunction (both symptomatic and asymptomatic) in patients undergoing treatment with anthracyclines depends critically on the timing of the initiation of cardioprotection therapy with betablockers and angiotensin-converting enzyme inhibitors.^48^

These findings indicate that a different approach to antitumor treatment-related cardiotoxicity is needed and we cannot anymore consider trastuzumab-related cardiotoxicity less important than anthracycline-related cardiotoxicity or only as a consequence of previous anthracycline treatment as it occurs also in trastuzumab monotherapy. Thus it becomes essential to identify patients with high risk factors or asymptomatic heart dysfunction before starting the treatment with trastuzumab, in order to treat them appropriately or strongly reduce the risk factors.

Considering these important observations, the type I and type II classification, based on reversible or not-reversible cardiotoxicity, has become inappropriate as the reversibility concept is ambiguous. It has been demonstrated that ErbB2 inhibitors can be toxic for cardiomyocytes when used in monotherapy in a fashion similar to doxorubicin.^39^ In particular trastuzumab induces and promotes activation of apoptotic pathway,^20,21,37^ thus triggering cell death. These effects may lead to cardiomyopathy or heart failure in treated patients as described above, especially in elder patients.^13-15,25^

The incidence of cardiotoxicity in the real-world population of cancer patients treated with trastuzumab monotherapy is completely different with respect to that observed in clinical trials, as adjuvant clinical trials of trastuzumab have typically enrolled younger women without cardiac comorbidities, excluding older people or people affected by cardiovascular diseases,^8,49,50^ thus leading to understimate the risk factors related to therapeutic protocol in clinical use based on trastuzumab monotherapy. Similarly, a recent meta-analysis study ^51^ showed that the combinatorial treatment of trastuzumab with the other FDA approved ErbB-targeted mAb Pertuzumab or TKI Lapatinib does not significantly increase the cardiac toxicity compared to that of single agents. However the same authors believe that appropriate patient selection is essential to prevent potential cardiac toxicity, and additional data should be collected in future trials to confirm these data.^51^

Furthermore, the incidence of Trastuzumab cardiotoxicity after ten years follow up is underrated, because it does not represent the incidence of real-world population of cancer patients treated with trastuzumab alone.

Since older patients are increasingly treated with these anti-ErbB2 drugs, and trastuzumab is widely used for its efficient therapeutic effects on metastatic cancer thus prolonging even more patient's survival in last decades, a different approach is needed to guarantee the survival of treated patients to avoid that women recovered from cancer paradoxically die for heart failure.

Also other immunotherapies and targeted drugs have been included in the type II cardiotoxicity such as angiogenesis inhibitors Bevacizumab (anti-VEGF humanized mAb), and TK inhibitors ^52^ even though the cardiotoxicity-associated mechanisms and clinical symptoms are very different. For istance, distruption of VEGFR-signaling leads to the reduction of capillary density causing contractile dysfunction, fibrosis and heart failure, whereas distruption of PDGFR-signaling triggers apoptosis and necrosis of the cardiac myocytes, or HIF-inhibition may impair myocardial response to acute or chronic ischemia.^52^ Thus, this general use of the term type II cardiotoxicity is not appropriate for most of the biologics.

On the basis of these considerations may we still consider Type I and Type II cardiotoxicities clearly different? We propose to find a new different classification for cardiotoxic side effects mainly based only on the types of drugs used in cancer therapy, so that we can discriminate the anthracycline-related cardiotoxicity triggered by anthracyclines or anthracycline-like drugs, ErbB2-related cardiotoxicity, due to trastuzumab or other ErbB2 inhibitors and anti-angiogenic – related cardiotoxicity (both antibodies and small molecule kinase inhibitors).
